# Five year neurodevelopment outcomes of perinatally HIV‐infected children on early limited or deferred continuous antiretroviral therapy

**DOI:** 10.1002/jia2.25106

**Published:** 2018-05-03

**Authors:** Barbara Laughton, Morna Cornell, Martin Kidd, Priscilla Estelle Springer, Els Françoise Marie‐Thérèse Dobbels, Anita Janse Van Rensburg, Kennedy Otwombe, Abdel Babiker, Diana M Gibb, Avy Violari, Mariana Kruger, Mark Fredric Cotton

**Affiliations:** ^1^ Family Clinical Research Unit Department of Paediatrics and Child Health Faculty of Medicine and Health Sciences Stellenbosch University Tygerberg Hospital Cape Town South Africa; ^2^ Centre for Infectious Disease Epidemiology and Research, and Division of Epidemiology and Biostatistics School of Public Health and Family Medicine Faculty of Health Sciences University of Cape Town Cape Town South Africa; ^3^ Centre for Statistical Consultation Department of Statistics and Actuarial Sciences University of Stellenbosch Matieland South Africa; ^4^ Perinatal HIV Research Unit Faculty of Health Sciences University of the Witwatersrand Johannesburg South Africa; ^5^ Medical Research Council Clinical Trials Unit University College London London United Kingdom

**Keywords:** HIV care continuum, Children, ARV, CHER trial, Early time‐limited antiretroviral therapy, Neurodevelopment, Treatment interruption, Griffiths mental development scales, Visual perception

## Abstract

**Introduction:**

Early antiretroviral therapy (ART) has improved neurodevelopmental outcomes of HIV‐infected (HIV‐positive) children; however, little is known about the longer term outcomes in infants commencing early ART or whether temporary ART interruption might have long‐term consequences. In the children with HIV early antiretroviral treatment (CHER) trial, HIV‐infected infants ≤12 weeks of age with CD4 ≥25% were randomized to deferred ART (ART‐Def); immediate time‐limited ART for 40 weeks (ART‐40W) or 96 weeks (ART‐96W). ART was restarted in the time‐limited arms for immunologic/clinical progression. Our objective was to compare the neurodevelopmental profiles in all three arms of Cape Town CHER participants.

**Methods:**

A prospective, longitudinal observational study was used. The Griffiths mental development scales (GMDS), which includes six subscales and a global score, were performed at 11, 20, 30, 42 and 60 months, and the Beery‐Buktenica developmental tests for visual motor integration at 60 months. HIV‐exposed uninfected (HEU) and HIV‐unexposed (HU) children were enrolled for comparison. Mixed model repeated measures were used to compare groups over time, using quotients derived from standardized British norms.

**Results:**

In this study, 28 ART‐Def, 35 ART‐40W, 33 ART‐96W CHER children, and 34 HEU and 39 HU controls were enrolled. GMDS scores over five years were similar between the five groups in all subscales except locomotor and general Griffiths (interaction *p* < 0.001 and *p* = 0.02 respectively), driven by early lower scores in the ART‐Def arm. At 60 months, scores for all groups were similar in each GMDS scale. However, Beery visual perception scores were significantly lower in HIV‐infected children (mean standard scores: 75.8 ART‐Def, 79.8 ART‐40W, 75.9 ART‐96W) *versus* 84.4 in HEU and 90.5 in HU (*p* < 0.01)).

**Conclusions:**

Early locomotor delay in the ART‐Def arm resolved by five years. Neurodevelopmental outcomes at five years in HIV‐infected children on early time‐limited ART were similar to uninfected controls, apart from visual perception where HIV‐infected children scored lower. Poorer visual perception performance warrants further investigation.

## 1 Introduction

HIV encephalopathy, a common finding in perinatally HIV‐infected children, has decreased since the introduction of combination antiretroviral therapy (ART) 1. Other neurodevelopmental deficits despite ART remain a concern. Although early viral suppression is beneficial for neurodevelopmental outcomes 2, 3, 4, 5, the long‐term outcomes of children after early ART are unclear and may be compromised by cumulative toxicity or waning adherence. Early treatment with planned interruptions after early virological suppression is a possible solution.

The Children with HIV Early Antiretroviral (CHER) trial conducted in South Africa (2005 to 2011) compared early time‐limited ART with deferred ART in asymptomatic HIV‐infected infants 6, 7. HIV‐infected infants with CD4 ≥25% were randomized to one of three strategies: (i) ART deferred until indicated (ART‐Def), (ii) early limited ART for 40 weeks (ART‐40W) and (iii) early limited ART for 96 weeks (ART‐96W). Continuous ART was initiated (in ART‐Def) and re‐initiated (in ART‐40W and ART‐96W) if CD4% declined <25% in the first year of life and <20% thereafter, or for protocol‐defined severe stage B or stage C clinical disease. After a median of 4.8 years, the superiority of early time‐limited over deferred continuous ART was confirmed 6.

A neurodevelopmental sub‐study of CHER found significantly better locomotor and general development scores at 11 months for early ART, with children in ART‐Def scoring significantly lower than the combined early ART arms. However, the early treatment arms had not yet interrupted ART 8. These neurodevelopmental outcomes contrast with older children from the PREDICT study which showed no difference between early *versus* deferred ART in children with CD4 15% to 24%. In PREDICT, HIV‐infected children performed worse than uninfected controls 9.

Here, we present the neurodevelopmental profiles over five years of this CHER sub‐study which includes data from the treatment interruption phase. We hypothesized: firstly that the early treatment arms would do better than the deferred arm, and that the improved neurodevelopmental outcomes seen at 11 months in the early treatment arms would persist despite time off therapy, and be similar to HIV‐uninfected controls; secondly that within the early treatment groups, carefully guided ART interruption would not affect neurodevelopmental outcomes.

## 2 Methods

### 2.1 Study Design and Participants:

This was a prospective, longitudinal observational sub‐study of CHER trial, conducted at the Cape Town site only, between 2006 and 2013. Of 411 infants enrolled onto CHER with CD4 ≥25%, 119 HIV‐infected infants in Cape Town were available, along with 42 perinatally HIV‐exposed uninfected (HEU) and 42 HIV‐unexposed (HU) controls from a concurrent linked vaccine study 10. Inclusion criteria for the sub‐study were: birthweight >2000 g, normal neurological examination at a clinical visit near three months of age, no dysmorphic syndromes or central nervous system (CNS) insults, for example foetal alcohol exposure, birth asphyxia or metabolic abnormalities. HEU and HU controls were enrolled at the same time to provide a reference group for interpreting neurodevelopmental scores within the socio‐economic and cultural context. HEU infants also controlled for ART exposure to prevent mother to child transmission (PMTCT) and circumstances surrounding growing up with an HIV‐infected mother. Mothers or legal guardians were approached from 2006 during a CHER or Vaccine study visit. Written consent was obtained in their preferred language. Participants who missed early enrolment were included at a later visit. The Stellenbosch University Health Research Ethics Committee approved the study, registration: N05/05/092. Due to attrition of controls, additional five year old Xhosa children (7 HEU, 3 HU) were enrolled from the original source communities in 2012. Eligibility criteria included documented evidence of mother's HIV‐negative status at birth, child's HIV‐1 antibody test negative, birthweight >2000 g, no history of CNS insults, being clinically healthy with normal medical history and general examination performed by a study doctor.

### 2.2 Neurodevelopmental Assessments:

The Griffiths Mental Development Scales (GMDS) were performed at a CHER study visit. The baby scales (0 to 2 years) were used at 11 and 20 months 11 and the extended revised version (2 to 8 years) at 30, 42 and 60 months 12. The GMDS assesses neurodevelopment on six subscales: locomotor, personal‐social, hearing and language, eye and hand coordination, and performance (visuospatial skills including speed and precision), adding practical reasoning after 24 months. A general Griffiths score, an average of the subscales, is also obtained.

One of the four GMDS‐trained paediatricians conducted the assessments, assisted by a GMDS‐trained translator using standardized translations, all blinded to treatment arm allocation. One paediatrician conducted only four assessments, all at 11 months. The other three assessed at all time points. At the beginning of each age time point, the paediatricians assessed a child together and marked independently, and compared scores and discussed discrepancies afterwards. This was repeated on different children until scoring was similar. The GMDS provides standardized norms from typical British children. Quotients were used to compare performance at different time points. The 0 to 2 year scale provides quotients from raw scores, and 2 to 8 year scales provide percentiles and z‐scores. For 2 to 5 year olds, we converted raw scores into age equivalents (from the standardized scores 50th percentiles) and calculated a quotient as a percentage of the child's chronological age, for the subscales and general Griffiths. At 60 months of age, the Beery‐Buktenica developmental tests of visual motor integration (Beery‐VMI), visual perception and motor coordination (sixth edition) were administered 13. The Beery‐VMI measures the ability to coordinate visual perceptual and motor skills by copying geometric forms. Visual perception requires the child to identify matching forms, and motor coordination involves drawing the forms by connecting dots and staying within paths provided, abilities that are not assessed in the GMDS. Standard scores were calculated from raw scores using USA norms. Neurodevelopmental scores are standardized with a mean (SD) quotient or standard score of 100 (15), scores of 90 to 109 are considered average and <70 as intellectual impaired/delayed 11, 13.

Neurological examination and head circumference measurement were performed at each visit and the Child Behavior Checklist completed at 20, 42 and 60 months (reported elsewhere) 14. Parents or caregivers received a written report after each assessment with advice for stimulating weaker areas of development. Children with suspected sensory deficits or significant developmental delay were referred to appropriate diagnostic and therapeutic services. We included assessments previously reported at 11 months 8 to better present the longitudinal developmental profile over five years. Primary endpoints were neurodevelopmental scores at each assessment.

### 2.3 Clinical data

On CHER, HIV‐infected participants had regular clinical visits: every 4 weeks until week 24, every 8 weeks until week 48 and then every 12 weeks. Assessments included: physical examination, ART adherence and management of any illnesses or adverse events, and T‐cell subsets were done. HIV‐RNA viral loads (VL) were only performed if treatment failure was suspected until November 2009, thereafter VL were performed 6 monthly. A blinded independent clinical endpoint review committee adjudicated CDC severe stage B and C events and HIV encephalopathy diagnosis without knowledge of CD4 values, ART status or treatment group 6, 7. HEU and HU children had three monthly clinical assessments for 18 months and then annually thereafter 10.

Demographic and clinical information were obtained from the CHER database. In HIV‐infected participants, viral load (VL) and CD4 values from baseline and within six months of the 60 months assessment were used, together with baseline CMV viraemia 15. HIV‐negative status of controls on the vaccine trial was confirmed between 16 and 28 weeks of age 10.

### 2.4 Statistical analysis

We used Statistica version 13 (software.dell.com. Dell Inc. 2015). The primary analysis compared neurodevelopmental quotients over time in five groups (three HIV‐infected: ART‐Def, ART‐40W and ART‐96W, and two uninfected: HEU, HU) using linear mixed models with group and time as categorical fixed effects, and participants as random effect. We opted for parsimony and therefore compound symmetry was used. Sample size calculations were based on predicted neurodevelopmental outcomes, which we expected to be larger. To detect an average effect size of 7.5 (half SD on GMDS and Beery tests) at 5% significance level, 30 per group would provide 90% power and 25 per group 80% power.

Due to concerns about using British norms for GMDS outcomes, we conducted *post‐hoc* analyses using age‐adjusted raw scores. Comparison of scores over 5 years was not possible, due to differences in raw score calculation <2 years and >2 years. We therefore compared, time points 1 to 2 and time points 3, 4 and 5 separately.

Summary statistics for background information were compared using chi‐squared test for categorical variables and one‐way analysis of variance (ANOVA) for continuous measures. Mean (SD) or median [IQR] were reported, depending on normality and homogeneity of variance.

Additional analysis was performed combining the three HIV‐infected arms. Spearman correlations were determined between neurodevelopmental outcomes at 60 months and the following: (i) age at starting ART, (ii) time on ART, (iii) time to VL suppression (age at first VL <400 copies/ml) and (iv) CD4 values at enrolment onto CHER. A 95% confidence interval was calculated and significance established at *p* < 0.05. For variables in the mixed model analysis, the Fisher least significant difference was used.

## 3 Results

### 3.1 Participants

Ninety‐six HIV‐infected infants of the 119 CHER trial participants in Cape Town were enrolled in the neurodevelopmental sub‐study: 28 ART‐Def (excluded two refusals and eight deaths before 11 months), 35 ART‐40W (excluded four refusals and two to foetal alcohol exposure (FAE)) and 33 ART‐96W (excluded four refusals and one glutaric‐aciduria, one FAE, one non‐adherence to CHER protocol); 63 of 84 uninfected controls from the vaccine study were enrolled; and 10 additional controls at 60 months (total enrolled: 34 HEU, 39 HU). One infant in ART‐96W died at 21 months (disseminated tuberculosis) and one HU child died at 48.5 months (motor vehicle accident). At 60 months, retention in arms was 26/28(93%) in ART‐Def, 29/35(83%) ART‐40W and 23/33(70%) ART‐96W, and retention in controls from baseline was 15/27(56%) in HEU and 25/36(69%) HU. For details on participants enrolled and assessed at each time point, see Figure S1.

### 3.2 Participant Characteristics

There were more boys in the controls than the HIV‐infected groups (Table 1). Birthweight was similar between groups. There was variability in the mode of delivery, with vaginal deliveries ranging from 68% (HEU) to 86% (ART‐40W). For PMTCT, most mothers and babies received nevirapine and zidovudine. Three HIV‐positive mother‐child dyads had no PMTCT. The mean age of mothers at birth of HEU infants was slightly older than the other groups (30.9 years *vs*. means between 27 and 28 years). Maternal education level was similar between groups. Almost all HIV‐infected infants, but only 36% of HU infants, were Xhosa‐speaking. In the HIV‐infected groups, baseline CD4 parameters and mean VL were similar while the proportion with CMV viraemia at baseline ranged from 13% to 29%.

**Table 1:** Demographic data and clinical characteristics of all study participants

	HIV infected			Uninfected		*p* value
	ART‐Def (n = 28)	ART‐40W (n = 35)	ART‐96W (n = 33)	HEU (n = 34)	HU (n = 39)	
Gender: Male	11 (39%)	15 (43%)	13 (40%)	19 (56%)	23 (59%)	0.29*
Birthweight, g, mean (SD)	3036 (407)	3096 (445)	2912 (411)	3090 (513)	3127 (561)	0.36*
Delivery						
NVD n (%)	20 (71%)	30 (86%)	28 (85%)	23 (68%)	31 (84%)	
C/S n (%)	8 (29%)	5 (14%)	5 (15%)	11 (32%)	6 (16%)	0.23*
Unknown					2	
PMTCT exposure						
Mother n (%)	27 (96%)	31 (89%)	31 (84%)	29 (85%)	–	0.14#
Child n (%)	25 (89%)	32 (91%)	32 (97%)	29 (85%)	–	0.15#
No PMTCT n	0	2	1	0		
Unknown n	0	0	0	5 (15%)		
Mother's age at birth (years) mean (SD)	27.2 (5.1)	27 (4.4)	27.2 (5.8)	30.9 (14.1)	28 (7.2)	0.26*
Mother's education mean (SD) (highest grade attended)	10.0 (2.1)	9.4 (2.3)	9.4 (2.7)	9.9 (2.4)	9.8 (2.0)	0.70*
Language						
Xhosa n (%)	27 (96%)	32 (91%)	30 (91%)	28 (88%)	14 (36%)	<0.001*
Afrikaans n	1	3	3	4	25	
English n				2		
Mean (SD) at baseline						
CD4 count (cells/μl)	1781 (672)	2082 (968)	2076 (1151)	–	–	0.39§
CD4%	35.8 (8.4)	35.5 (8.6)	34.5 (8.6)			0.81§
Viral load at baseline copies/ml Mean (SD)	598,504 (243,254)	528,435 (250,021)	593,933 (244,958)	–	–	0.38§
CMV PCR at baseline n (%) >25 copies/ml	5/25 (20%)	8/28 (29%)	4/30 (13%)	–	–	0.35§

Treatment groups: ART‐Def: ART deferred until symptomatic, ART‐40W: early ART until 40 weeks then planned interruption, ART‐96W: early ART until 96 weeks then planned interruption. Control groups: HEU: HIV‐exposed uninfected, HU: HIV‐unexposed. Chi‐square for categorical variables and ANOVAs for continuous variables

*p* values: *between 5 groups, ^#^between 4 groups, ^§^between 3 groups.

Of HIV‐infected participants enrolled, those developing CDC stage severe B or stage C diagnosis were 10/28(36%) in ART‐Def, 15/35(43%) in ART 40W and 11/33(33%) in ART 96W. These included HIV encephalopathy in four (14%) ART‐Def (diagnosed at 5, 9, 31 and 61 months), five (14%) ART‐40W infants (at 19, 20, 21, 24 and 31 months) and two (6%) ART‐96W (at 10 and 31 months). Table 2 further describes characteristics of HIV‐infected participants.

**Table 2:** Descriptive characteristics of HIV‐infected participants

Antiretroviral therapy for all enrolled participants				
	ART‐Def	ART‐40W	ART‐96W	*p* value
	N = 28	N = 35	N = 33	
Age at ART initiation, months mean (SD)	6.8 (3.5)	1.3 (0.5)	1.5 (0.5)	<0.001
Treatment interruption: n (%)	Not applicable	29/35 (88%)	20/33 (61%)	0.04
Median [IQR] time interrupted (months)		7 [5 to 11]^a^	8 [7 to 36]^b^	

Assessments at each time in time‐limited treatment groups and proportion off ART				
	ART 40‐W		ART‐96W	
Assessment age	Number assessed	Number interrupted (%)	Number assessed	Number interrupted (%)
11 months	34	0	32	0
20 months	33	14 (42%)	29	0
30 months	32	5 (16%)	28	16 (57%)
42 months	29	1 (4%)	26	6 (23%)
60 months	29	1 (4%)	23	5 (22%)

Antiretroviral therapy and clinical parameters at five years for those assessed at 5 years				
	ART‐Def	ART‐40W	ART‐96W	*p* value
	N = 26	N = 29	N = 23	
Cumulative time on ART at 5 years. assessment, (months) mean (SD)	54.6 (3.6)	49.4 (12)	48.1 (14.5)	<0.001
Severe stage B/C diagnosis, n (%)	9 (35%)	13 (45%)	6 (26%)	>0.2^c^
HIV encephalopathy, n (%)	4 (15%)	4 (14%)	2 (9%)	>0.5 c
CD4 count mean (SD)	1080 (363)	1192 (492)	1032 (541)	0.45
CD4% mean (SD)	36.6 (5.8)	34.4 (6.9)	33.1 (8.2)	0.22
VL <400 copies/ml	24/26 (92%)	28/29 (97%)^a^	17/23 (74%)^b^	
WHO height‐for‐age z‐score mean (SD)	−0.64 (1.0)	−0.90 (1.2)	−0.83 (0.8)	0.59
WHO weight‐for‐age z‐score mean (SD)	0.09 (1.1)	0.05 (1.1)	−0.26 (0.8)	0.44

Treatment groups: ART‐Def: ART deferred until symptomatic, ART‐40W: early ART until 40 weeks then planned interruption, ART‐96W: early ART until 96 weeks then planned interruption.

a Includes 1 who had not yet restarted ART at 60 months.

b Includes 5 who had not yet restarted ART at 60 months and one left study before restarting.

c Pairwise chi‐square comparison between groups.

### 3.3 Neurodevelopmental assessments and outcomes:

**Figure 1:**
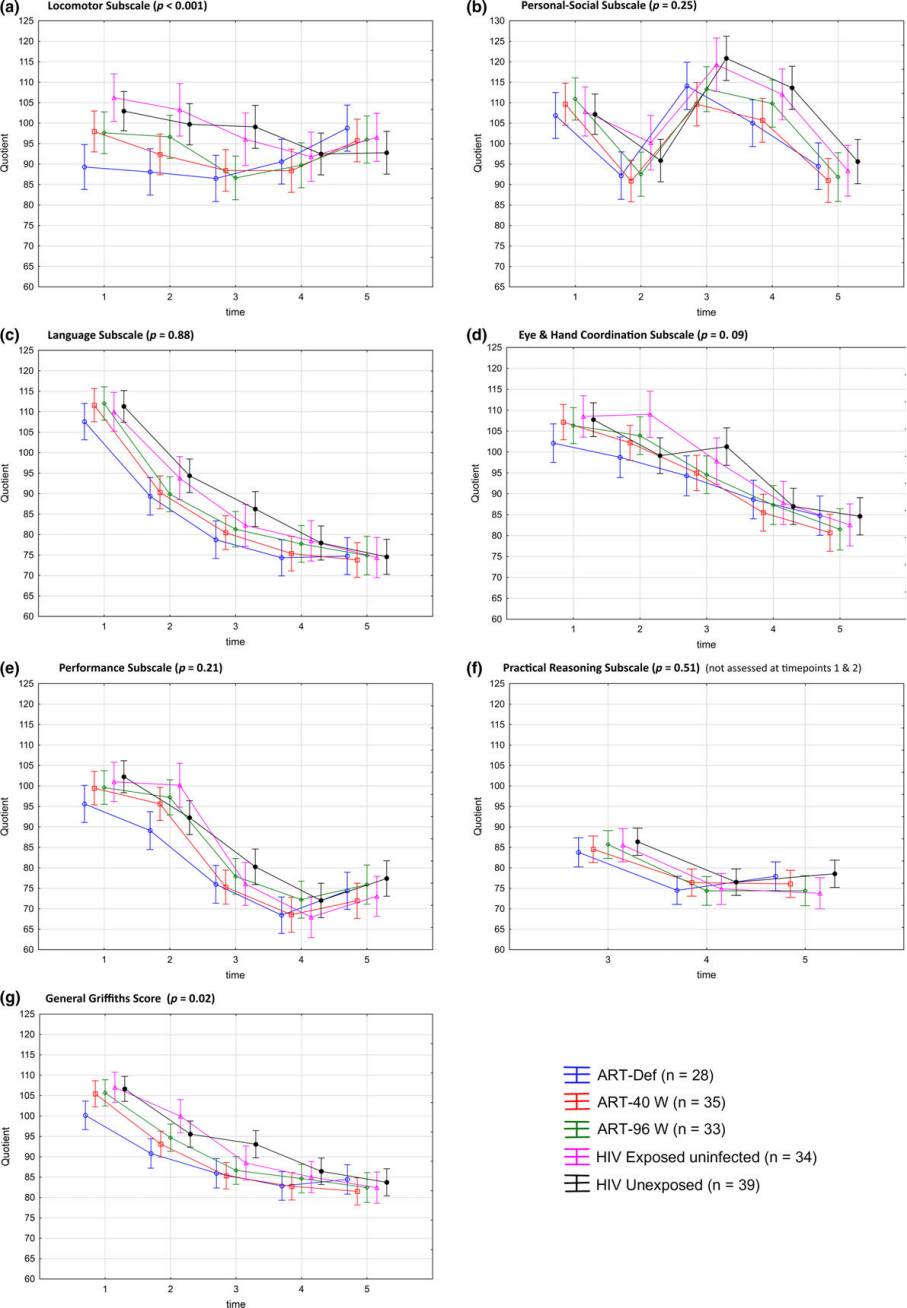
Comparison of Griffiths scales of mental development scores over time per arm. LS means, Type III decomposition, Vertical bars denote 0.95 confidence intervals, interaction p‐values. X Axis: Mean age at time points: 1 = 11 months, 2 = 20 months, 3 = 30 months, 4 = 42 months, 5 = 60 months. Note: Lines connecting scores added for visual clarity and do not imply participants identical at each time point.

In general, mean GMDS scores declined over time in all subscales and in all groups, including controls. The most significant differences in longitudinal profile between groups were in the locomotor subscale (*p* < 0.001) and general Griffiths scale (*p* = 0.02), driven by initial lower scores in ART‐Def arm at 11 and 20 months. The control groups had higher scores than the HIV‐infected arms at the first three assessments in most subscales. However, scores for all groups were similar at 42 and 60 months, with mean quotient for each group ranging from 93.2 to 98.7 for the locomotor subscale and from 81.8 to 84.7 for general Griffiths (Figure 1 and Table 3). Not all children were assessed at each time point due to missed visits or withdrawal, mainly relocation to rural areas.

**Table 3:** Locomotor subscale and general Griffiths: summary of scores at each time point and comparison between groups

Locomotor subscale: descriptive statistics for each group at each time point					
Assessment age	1. ART‐Def	2. ART‐40W	3. ART‐96W	4. HEU	5. HU
11 months	n = 27	n = 32	n = 32	n = 23	n = 35
Mean quotient ±SD	89.5 ± 16.1	98.0 ± 11.8^a^	97.6 ± 14.7^a^	105.9 ± 15.7	102.5 ± 12.9
95% CI	(83.1 to 95.8)	(93.8 to 102.2)	(92.1 to 102.9)	(99.1 to 112.6)	(98.0 to 106.9)
20 months	n = 25	n = 33	n = 29	n = 18	n = 31
Mean quotient ±SD	87.7 ± 18.7	92.6 ± 17.4	97.0 ± 14.0	103.6 ±9.4	99.8 ± 12.3
95% CI	(80.0 to 95.4)	(86.4 to 98.8)	(91.7 to 102.4)	(98.9 to 108.3)	(95.3 to 104.4)
30 months	n = 25	n = 31	n = 28	n = 19	n = 28
Mean quotient ±SD	86.1 ± 16.4	89.2 ± 15.2^a^	87.0 ± 13.3	95.7 ± 14.2^a^	99.2 ± 14.3
95% CI	(79.3 to 92.8)	(83.6 to 94.7)	(81.8 to 92.1)	(88.6 to 102.8)	(93.7 to 104.8)
42 months	n = 27	n = 29	n = 26	n = 21	n = 30
Mean quotient ±SD	90.7 ± 14.5	89.2 ± 14.4^a^	90.1 ± 13.4	91.2 ± 12.8	91.8 ± 16.6
95% CI	(85.0 to 96.4)	(83.6 to 94.7)	(84.7 to 95.5)	(85.3 to 97.0)	(85.6 to 98.0)
60 months	n = 26	n = 29	n = 23	n = 22	n = 28
Mean quotient ±SD	98.7 ± 14.8^a^	96.3 ± 18.0	96.7 ± 13.0	97.1 ± 11.5	93.2 ± 13.2
95% CI	(92.6 to 104.8)	(89.5 to 103.2)	(91.0 to 102.3)	(92.0 to 102.2)	(88.1 to 98.3)

*p*‐values for pairwise comparisons between groups for locomotor quotients.																				
Assessment age	1 *versus* 2			1 *versus* 3		1 *versus* 4		1 *versus* 5		2 *versus* 3		2 *versus* 4		2 *versus* 5		3 *versus* 4		3 *versus* 5		4 *versus* 5
11 months	0.02*			0.03*		<0.001*		<0.001*		0.9		0.03*		0.15		0.03*		0.14		0.39
20 months	0.26			0.03*		0.001*		0.003*		0.24		0.01*		0.04*		0.12		0.41		0.4
30 months	0.61			0.97		0.03*		0.001*		0.63		0.07		0.004*		0.03*		0.001*		0.47
42 months	0.56			0.82		0.77		0.62		0.73		0.4		0.27		0.61		0.47		0.87
60 months	0.44			0.5		0.59		0.12		0.96		0.85		0.43		0.9		0.42		0.35

General Griffiths scale: descriptive statistics for each group at each time point					
Assessment	1. ART‐Def	2. ART‐40W	3. ART‐96W	4. HEU	5. HU
11 months	n = 27	n = 32	n = 32	n = 23	n = 35
Mean quotient ±SD	100.2±13.4	105.5±10.5^b^	105.6±10.2	107.3±10.0	106.5±11.5
95% CI	(94.9 to 105.5)	(101.7 to 109.3)	(101.9 to 109.3)	(103.0 to 111.6)	(102.6 to 110.5)
20 months	n = 25	n = 33	n = 29	n = 18	n = 31
Mean quotient ±SD	90.4±13.6	93.3 to 12.4	94.9±9.1	101.1±8.0	95.6±11.0
95% CI	(84.8 to 96.0)	(88.9 to 97.7)	(91.5 to 98.4)	(97.2 to 105.1)	(91.5 to 99.6)
30 months	n = 25	n = 31	n = 28	n = 19	n = 28
Mean quotient ±SD	85.6±6.0	85.6±7.2^a^	86.7±6.9	89.0±7.5^b^	93.5±9.9
95% CI	(83.1 to 88.1)	(82.9 to 88.2)	(84.1 to 89.4)	(85.2 to 92.9)	(89.6 to 97.3)
42 months	n = 27	n = 29	n = 26	n = 21	n = 30
Mean quotient ±SD	83.0±6.9	83.3±5.9^a^	84.7±8.4	84.8±7.8	86.0±10.1
95% CI	(80.2 to 85.7)	(81.0 to 85.6)	(81.4 to 88.1)	(81.2 to 88.4)	(82.3 to 89.8)
60 months	n = 26	n = 29	n = 23	n = 22	n = 28
Mean quotient ±SD	84.3±6.3^a^	81.8±7.0	82.6±6.5	82.8±8.4^a^	84.7±7.7
95% CI	(81.7 to 86.9)	(79.1 to 84.5)	(79.8 to 85.4)	(79.0 to 86.6)	(81.7 to 87.7)

*p*‐values for pairwise comparisons between groups for general Griffiths quotients										
Assessment age	1 *versus* 2	1 *versus* 3	1 *versus* 4	1 *versus* 5	2 *versus* 3	2 *versus* 4	2 *versus* 5	3 *versus* 4	3 *versus* 5	4 *versus* 5
11 months	0.03*	0.02*	0.008*	0.006*	0.92	0.52	0.59	0.59	0.67	0.88
20 months	0.35	0.12	0.001*	0.05	0.5	0.01*	0.28	0.05	0.71	0.1
30 months	0.8	0.78	0.37	0.005*	0.58	0.25	0.001*	0.51	0.01*	0.09
42 months	0.97	0.47	0.41	0.14	0.44	0.38	0.12	0.89	0.47	0.58
60 months	0.24	0.45	0.45	0.78	0.71	0.72	0.35	1.0	0.61	0.61

^a^1 participant and ^b^2 participants did not have usable scores (refused too many items or tester error). *Significant differences.

Results for raw scores (controlling for age) were similar to those for quotients. Differences in developmental profile between groups in the locomotor subscale were confirmed between 30, 42 and 60 months (interactive *p* = 0.009), but the general Griffiths was not as robust (interactive = *p* 0.1). The raw scores also confirmed significant differences in pairwise comparisons between groups in early assessments, and no difference between arms at 5 years, observed for comparing the quotients. Analysis of raw scores in other Subscales confirmed no difference in profiles. (See Supplementary material Tables S3, S4 and S5).

For visual perception at 60 months, the HIV‐infected groups scored 5 to 9 points lower than HEU group and 10 to 14 points lower than HU, but Beery‐VMI and motor coordination were similar (Table 4). During assessments, no children were suspected of having visual problems.

**Table 4:** Descriptive statistics for scores on the Beery‐Buktenica developmental tests for each group

Assessment	1. ART‐Def n = 26	2. ART‐40W n = 29	3. ART‐96W n = 22	4. HEU n = 21	5. HU n = 28	*p* value
Visual motor integration (VMI)						
Mean quotient ±SD	89.7 ± 9.0	89.0 ± 7.3	90.3 ± 11.7	88.0 ± 10.3	92.7 ± 10.5	0.89
95% CI	(86.1 to 93.3)	(86.2 to 91.8)	(85.2 to 95.4)	(83.3 to 92.7)	(88.6 to 96.7)	
Visual perception						
Mean quotient ±SD	75.8 ± 15.9^a^	79.8 ± 14.7^a^	75.9 ± 13.5	84.4 ± 13.5^b^	90.5 ± 9.3	<0.01
95% CI	(69.2 to 82.3)	(74.1 to 85.5)	(69.9 to 81.9)	(77.9 to 90.9)	(86.9 to 94.1)	
Motor coordination						
Mean quotient ±SD	94.1±10.6	93.6±8.4	96.5±7.3	93.9±12.3	92.9±12.3	0.8
95% CI	(89.8 to 98.3)	(90.4 to 96.8)	(93.3 to 99.7)	(88.3 to 99.5)	(88.1 to 97.7)	

*p*‐values for pairwise comparisons between groups for Beery‐Buktenica developmental tests										
Assessment	1 *versus* 2	1 *versus* 3	1 *versus* 4	1 *versus* 5	2 *versus* 3	2 *versus* 4	2 *versus* 5	3 *versus* 4	3 *versus* 5	4 *versus* 5
Visual motor integration	0.78	0.83	0.56	0.26	0.62	0.73	0.15	0.44	0.39	0.1
Visual perception	0.28	0.98	0.04*	<0.001*	0.31	0.26	0.004*	0.05*	<0.001*	0.13
Motor coordination	0.87	0.42	0.96	0.68	0.33	0.92	0.79	0.41	0.23	0.74

Treatment groups: ART‐Def: ART deferred until symptomatic, ART‐40W: early ART until 40 weeks then planned interruption, ART‐96W: early ART until 96 weeks then planned interruption. Control groups: HEU: HIV‐exposed uninfected, HU: HIV‐unexposed.

^a^1 participant and ^b^2 participants did not complete the test. *Significant differences.

### 3.4 Antiretroviral therapy and responses:

Children on early ART began first‐line therapy (zidovudine, lamivudine and lopinavir/ritonavir) at a mean±SD of 1.3 ± 0.5 months (ART‐40W) and 1.5 ± 0.5 months (ART‐96W) compared to ART‐Def (6.8 ± 3.5 months; range 3.9 to 17.7) (Table 2). For ART‐40W, of 35 enrolled, two left the study before 40 weeks and 4 met criteria for not interrupting; 29/33(88%) interrupted at a mean age of 11.4 months for a median of 7.0 [IQR 5.0 to 11.0] months. At 60 months, one was still off ART. For ART‐96W, of 33 enrolled, three left the study, one died before 96 weeks and nine met criteria for not interrupting; 20/29(69%) interrupted treatment at a mean age of 24.5 months for a median of 8.0 [IQR 7.0 to 36.0] months (one withdrew before restarting). At 60 months, 14 had restarted and five were still off ART. The mean±SD cumulative time on ART until 60 months was similar among ART‐40W and ART‐96W and shorter than ART‐Def. One child changed to second line ART at 2 years due to CD4 <20% (in ART‐40W). Table 2 describes the proportion of participants off ART of those assessed at each assessment.

### 3.5 Clinical measures at five years

The proportion of infants with severe CDC stage B or C and HIV encephalopathy of those assessed at 5 years is described in Table 2. At 60 months, 92% in ART‐Def, 97% in ART‐40W and 74% in ART‐96W had VL <400 copies/ml. Mean CD4 parameters and mean WHO height‐for‐age z‐scores were similar in the groups.

### 3.6 Correlations between clinical parameters and neurodevelopment at five years

Combining all HIV‐infected infants, correlations were weak between neurodevelopmental outcomes at five years and age starting ART, baseline CD4 count, time on ART and time to first VL suppression (Spearman r between −0.19 and 0.08). Higher baseline CD4% correlated significantly with lower locomotor motor scores (r −0.23; *p* = 0.03) and marginally with Beery‐VMI (r −0.2; *p* = 0.06) and motor coordination (r −0.1; *p* = 0.06). (Supplementary Material Tables S1 and S2).

## 4 Discussion

Through serial testing, we demonstrated poorer locomotor and general Griffiths scores in ART‐Def at 11 and 20 months of age, which had resolved by 42 months and was maintained at five years. The limited differences between groups over time on personal‐social, language, eye and hand coordination, performance and practical reasoning GMDS subscales is encouraging. Our findings suggest that limited ART interruption under careful clinical guidance in asymptomatic children starting ART ≤12 weeks of age did not negatively affect their neurodevelopmental outcomes at five years. This supports clinical, immunological and virological findings from the larger CHER cohort that early time‐limited ART appears safe 6.

At 11 months, neurodevelopmental scores of ART‐Def were generally lower than the other arms. Our longer term findings provide new evidence that catch‐up/recovery is possible after delayed ART initiation. However, eight early deaths in ART‐Def prior to GMDS assessments may have contributed to a survivor effect.

Our finding that all HIV‐infected arms had visual perceptual scores significantly below HU children at 5 years, is concerning. Similarly, two HIV‐infected arms (ART‐Def and ART‐40W) also scored significantly below HEU children. This was despite viral suppression and suggests that HIV adversely affects visual perception regardless of ART strategy. However, cultural differences in early childhood stimulation may have influenced these findings as there were more Afrikaans‐speaking children in the HU group. This observation requires further investigation as visual perception may be the underlying cause of deficits found in later childhood. Visual perception involves recognition and discrimination of visual shapes and objects. This process involves the cognitive components and executive tasks of visual attention, memory, discrimination and imagery. Perceptual identification is processed by the ventral stream pathway 16, which may be vulnerable to HIV. Deficits may impair reading, writing and mathematics achievement 17. Deficits in visual spatial organization, processing and working memory have been described in HIV‐infected children, even in the context of normal cognitive development, higher CD4 counts or clinical stability 18, 19, 20, 21. Interestingly, there was no difference between groups on the Beery‐VMI and motor coordination tests. Abnormal visual perception in children with normal Beery‐VMI scores has been described 17. It is important to consider that the Beery‐VMI may not be sensitive enough to measure the specific visual perceptual deficit caused by HIV in young children.

It is possible that ART interruption at 96 weeks may be better than at 40 weeks, as suggested in the main results paper 6. There was more HIV encephalopathy in ART‐Def and ART‐40W compared to ART‐96W, and ART‐40W also had more CDC severe stage B and stage C diagnoses than the other arms, with the increase occurring largely between the 11 and 18 months' assessments during treatment interruption. On the other hand, fewer children underwent interruption or had baseline CMV viraemia in ART‐96W. Interpreting the effects of treatment interruption is difficult as some children randomized to ART‐40W or ART‐96W were not interrupted, there was a wide range of time off ART, and ART‐Def had a longer mean time on ART than the early time‐limited arms.

On secondary analysis there were no significant correlations between neurodevelopmental outcome at five years and the following parameters: age at starting ART, time to first viral suppression and time on ART. Interestingly, a higher CD4% at baseline significantly correlated with lower locomotor scores (Spearman r of −0.23) and marginally correlated with lower Beery‐VMI and motor coordination scores. Although counter‐intuitive, we considered a number of explanations. A higher CD4% in ART‐Def may have led to a longer period of observation prior to ART initiation; those on time‐limited ART (ART‐40W and ART‐96W) may have been more likely to interrupt ART than in those who reached an endpoint prior to interruption so would have remained on continuous ART. This issue requires further exploration.

It is interesting to note that on the locomotor subscale, the mean quotients decreased over time in both uninfected groups while increasing in HIV‐infected arms. Similar decline over time was noted in most other subscales. The ART‐Def and ART‐40W arms may be demonstrating catch up due to longer time on suppressive ART from an early age. However, this should be interpreted with caution due to small numbers in infected arms and the enrolment of additional controls at five years.

The GMDS may be insufficiently sensitive to discriminate between the groups at five years, thus creating an impression of “catch up” in the infected groups. Alternatively, the effects of poverty and deprivation on early childhood development may outweigh those of the different ART strategies on neurodevelopment 22, 23.

The decline in all GMDS scores except for the personal‐social subscale most likely reflects the application of British norms 11, 12 to South African children from impoverished environments, and emphasizes the importance of having uninfected controls from the same communities. The GMDS has Xhosa and Afrikaans translations, is widely used in South Africa and thought to be culturally fair 24, 25, 26. However, caution should be used when interpreting these results for clinical purposes. In a *post‐hoc* analysis, the longitudinal profiles of age‐adjusted raw scores supports our findings when using quotients for locomotor; however, there is a weaker association with general Griffiths and similar scores between groups at five years. We did not see a decline in scores over time for raw scores. (See supplemental material Tables S4 and S5)

The strengths of the study are that this is a relatively cohesive sample recruited from communities with minimal prenatal substance abuse and includes uninfected children from the same communities.. The similar demographic and clinical characteristics in the HIV‐infected arms reflect successful randomization of this sub‐study within the main trial. A further strength is the longitudinal nature of assessments, despite relatively small numbers and some attrition.

### 4.1 Limitations

Our results should be considered within the context of small numbers and variability between groups. The original sample size for this study was calculated assuming a larger difference in neurodevelopmental scores between groups and larger group numbers. Some missed visits and attrition further reduced sample sizes. An inherent bias was that controls had less clinical contact time than HIV‐infected children as they had no medication visits, and additional controls were added at five years. Although there were more Afrikaans‐speaking HU participants, they were from similar socio‐economic backgrounds to the Xhosa‐speaking participants with similar neurodevelopmental trajectories. However, we cannot rule out the cultural effects of early childhood rearing practices which may have influenced some comparisons. While we acknowledge that illnesses and maternal depression are important influences on early childhood development, we exclude the effects of hospitalizations and maternal depression due to small sample sizes relative to the number of arms and assessment time points. We have previously described the high incidence of maternal depression and effects of maternal trauma in this cohort 14, 27. As we did not determine whether the HIV infection occurred *in utero* or perinatally, each arm had potential heterogeneity, making further interpretation of ART interruption difficult.

Nevertheless, we found that early ART improved neurodevelopmental outcomes. Planned treatment interruption appeared safe in children who suppressed early. This is reassuring for situations where ART interruption is unavoidable, for example lack of supplies or social/political disruption.

All HIV‐infected children should be assessed for visual perceptual deficits and referred for intervention as needed, to improve educational outcomes.

## 5 Conclusions

HIV‐infected children on ART‐Def arm had locomotor delay at younger ages, which recovered by 5 years. For children with perinatal HIV infection, the neurodevelopmental outcomes at five years of asymptomatic children with preserved CD4 T‐cell percentages and receiving early but limited ART under strict clinical guidance, is similar to HIV‐uninfected neighbourhood controls. However, poorer visual perception in HIV‐infected children, irrespective of ART strategy, requires further exploration.

## Competing interests

The authors declare that they have no competing interests.

## Authors' contributions

AV, MFC, DMG and AGB designed the CHER trial and provided guidance to BL for design of neurodevelopmental sub‐study. BL and PES performed the neurodevelopmental assessments. EFMD provided clinical care to participants, AJvR was the study coordinator, AGB and KO were statisticians for the CHER trial and MK was the statistician for the neurodevelopmental sub‐study. BL and MC wrote the first draft of the paper and all co‐authors contributed to interpreting findings and writing the manuscript.

## Supporting information


**Figure S1.** Flow diagram of participants enrolled and assessments performed at each age per group.
**Table S1.** Correlation between time to first viral suppression (age at first viral load <400 copies/ml) and neurodevelopmental scores at 5 years
**Table S2.** Correlation between baseline CD4 values and neurodevelopmental scores at 5 years
**Table S3.** Comparison of statistical analysis using Quotients from British standardized norms and raw scores. Linear mixed model with group and time as categorical fixed effects: interaction *p* value
**Table S4.** Pairwise comparisons between groups of means for age‐adjusted raw scores and quotients in each group for general Griffiths scale
**Table S5.** Pairwise comparisons between groups of means for age‐adjusted raw scores and quotients in each group for locomotor subscale
