# Supplementary material for: Five year neurodevelopment outcomes of perinatally HIV‐infected children on early limited or deferred continuous antiretroviral therapy
Source: J Int AIDS Soc. 2018 May 3;21(5):e25106. doi: 10.1002/jia2.25106 (PMC5932637; doi:10.1002/jia2.25106)
Supplement: Supplementary file 1 — Figure S1. Flow diagram of participants enrolled and assessments performed at each age per group. Table S1. Correlation between time to first viral suppression (age at first viral load <400 copies/ml) and neurodevelopmental scores at 5 years Table S2. Correlation between baseline CD4 values and neurodevelopmental scores at 5 years Table S3. Comparison of statistical analysis using Quotients from British standardized norms and raw scores. Linear mixed model with group and time as categorical fixed effects: interaction p value Table S4. Pairwise comparisons between groups of means for age‐adjusted raw scores and quotients in each group for general Griffiths scale Table S5. Pairwise comparisons between groups of means for age‐adjusted raw scores and quotients in each group for locomotor subscale [file JIA2-21-e25106-s001.docx]

**Additional material for manuscript:**

**Five year Neurodevelopment Outcomes of perinatally HIV-infected children on early limited or deferred continuous antiretroviral therapy**


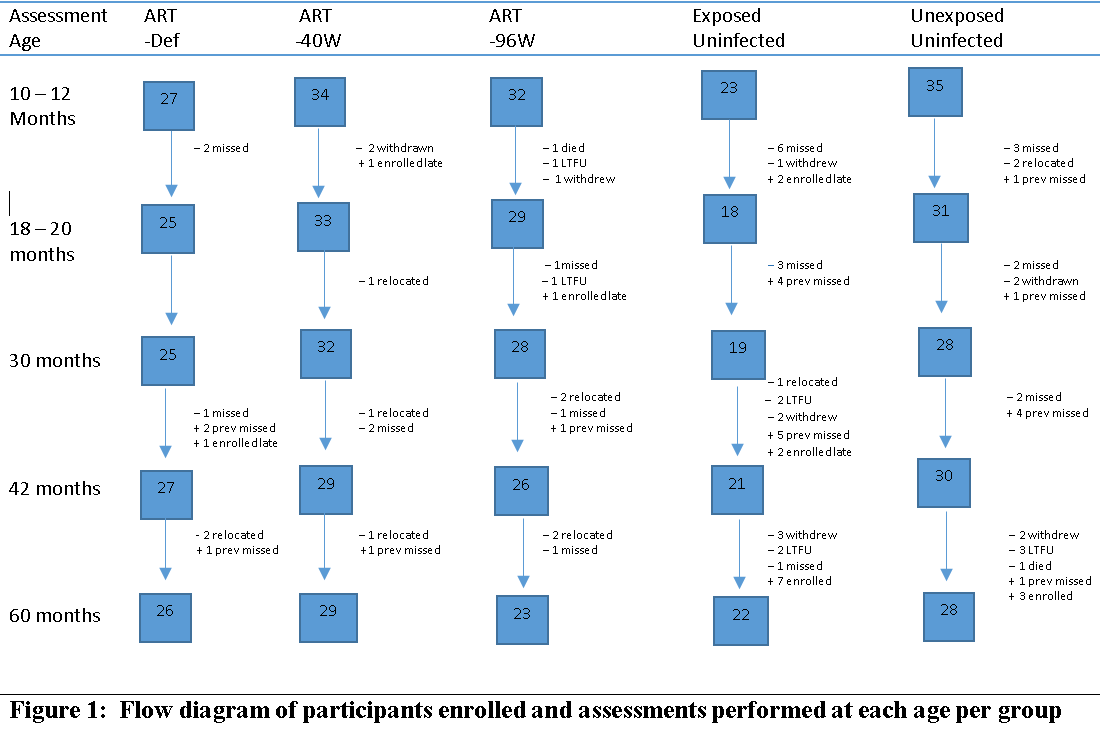


**Correlations between clinical parameters and neurodevelopment at 5 years:**

Age starting ART and the General Griffiths quotient at 5 years*:* Spearman r= -0·03

Time spent on ART and the General Griffiths quotient at 5 years: Spearman r=-0·13

Time to first HIV viral suppression and neurodevelopmental scores at 5 years: Spearman r ranged from -0·12 to 0·07 (table 1)

Baseline CD4 percentage and count and neurodevelopmental scores at 5 years: Spearman r ranged from -0·23 to 0·08 (table 2)

| **Table 1: Correlation between time to first viral suppression (age at first viral load <400 copies/ml) and neurodevelopmental scores at 5 years:** | | |
| --- | --- | --- |
|  | Spearman r | p value |
| **Griffiths Mental Development Scales:** | |  |
| Locomotor | 0.02 | 0.86 |
| Personal-Social | -0.1 | 0.4 |
| Language | -0.04 | 0.74 |
| Eye& Hand Coordination | 0.02 | 0.86 |
| Performance | -0.12 | 0.29 |
| Practical Reasoning | 0.05 | 0.65 |
| General Griffiths | -0.03 | 0.82 |
|  |  |  |
| **Beery-Buktenica Tests:** |  |  |
| Visual Motor Integration | -0.06 | 0.6 |
| Visual Perception | 0.04 | 0.75 |
| Motor Coordination | 0.07 | 0.55 |

| **Table 2: Correlation between baseline CD4 values and neurodevelopmental scores at 5 years** | | | | | |
| --- | --- | --- | --- | --- | --- |
|  | CD4 count | |  | CD4 % | |
|  | Spearman r | p value |  | Spearman r | p value |
| **Griffiths Mental Development Scales:** |  |  |  |  |  |
| Locomotor | -0.19 | 0.08 |  | -0.23 | 0.03 |
| Personal-Social | 0.00 | 0.99 |  | 0.08 | 0.48 |
| Language | 0.08 | 0.48 |  | -0.18 | 0.11 |
| Eye& Hand Coordination | -0.08 | 0.48 |  | -0.07 | 0.52 |
| Performance | -0.03 | 0.75 |  | -0.10 | 0.34 |
| Practical Reasoning | -0.09 | 0.41 |  | -0.03 | 0.79 |
| General Griffiths | -0.13 | 0.24 |  | -0.16 | 0.14 |
|  |  |  |  |  |  |
| **Beery-Buktenica Tests:** |  |  |  |  |  |
| Visual Motor Integration | -0.13 | 0.25 |  | -0.20 | 0.06 |
| Visual Perception | -0.19 | 0.08 |  | -0.07 | 0.53 |
| Motor Coordination | -0.10 | 0.37 |  | -0.21 | 0.06 |

**Comparison of statistical analysis using Quotients from UK standardised norms and raw scores.**

Raw scores were adjusted for age at each time point.

The raw scores derived from the Griffiths 0-2 year old (baby scales) are different to the Griffiths 2-5 year old (Extended Revised version) and it was not possible to compare raw scores over 5 time points. (Study objective was to compare neurodevelopmental profiles over 5 year of 5 groups of children). Analysis or raw scores was restricted to comparing the profile in the arms in two sections between time points 1-2 and between time points 3,4,5.

**Table 3. Comparison of statistical analysis using Quotients from British standardised norms and raw scores. Linear mixed model with group and time as categorical fixed effects: interaction p value**

| **Statistical method used** | **Raw scores with age as covariate** | | **Quotients** |
| --- | --- | --- | --- |
| Time points included | 1-2 | 3,4,5 | 1,2,3,4,5 |
| General Griffiths | 0.84 | 0.10 | 0.02 |
| Locomotor | 0.3 | 0.009 | <0.001 |
| Personal-Social | 0.37 | 0.67 | 0.25 |
| language | 0.15 | 0.67 | 0.88 |
| Eye& Hand Co-ordination | 0.40 | 0.46 | 0.09 |
| Performance | 0.40 | 0.88 | 0.21 |
| Practical Reasoning | n/a | 0.41 | *0.51 |

* only tested at 3,4,5

For the General Griffiths raw scores, the differences in neurodevelopmental trajectories in the arms is no longer significant.

The General Griffiths raw score is calculated differently at 1-2 (total of subtest raw scores) and 3,4,5 (average of subtest raw scores).

**Table 4; Pairwise comparisons between groups of means for age-adjusted raw scores and quotients in each group for general Griffiths scale.**

| Time point | Analysis method | ART-Def  Vs  ART-40W | ART-Def  vs  ART -96W | ART-Def  vs  HEU | ART-Def  vs  HU | ART-40W vs  ART-96W | ART-40W vs  HEU | ART-40W vs  HU | ART-96W vs  HEU | ART-96W vs  HU | HEU  vs  HU |
| --- | --- | --- | --- | --- | --- | --- | --- | --- | --- | --- | --- |
|  | **General Griffiths Scale** | | | | | | | | | | |
| 1 | Raw | 0.12 | 0.07 | 0.01 | 0.04 | 0.75 | 0.23 | 0.57 | 0.37 | 0.82 | 0.47 |
|  | Quotient | 0.03 | 0.02 | 0.008 | 0.006 | 0.92 | 0.52 | 0.59 | 0.59 | 0.67 | 0.88 |
| 2 | Raw | 0.51 | 0.10 | 0.00 | 0.14 | 0.27 | 0.01 | 0.36 | 0.14 | 0.9 | 0.1 |
|  | Quotient | 0.35 | 0.12 | 0.001 | 0.05 | 0.5 | 0.01 | 0.28 | 0.05 | 0.71 | 0.1 |
| 3 | Raw |  |  | 0.47 | 0.02 | 0.85 | 0.33 | 0.01 | 0.43 | 0.02 | 0.15 |
|  | Quotient | 0.8 | 0.78 | 0.37 | 0.005 | 0.58 | 0.25 | 0.001 | 0.51 | 0.01 | 0.09 |
| 4 | Raw | 0.90 | 0.38 | 0.48 | 0.11 | 0.44 | 0.55 | 0.13 | 0.89 | 0.49 | 0.42 |
|  | Quotient | 0.97 | 0.47 | 0.41 | 0.14 | 0.44 | 0.38 | 0.12 | 0.89 | 0.47 | 0.58 |
| 5 | Raw | 0.06 | 0.26 | 0.62 | 0.70 | 0.7 | 0.18 | 0.11 | 0.55 | 0.43 | 0.88 |
|  | Quotient | 0.24 | 0.45 | 0.45 | 0.78 | 0.71 | 0.72 | 0.35 | 1 | 0.61 | 0.61 |

For the pairwise comparisons of General Griffiths score, there are discrepancies between the raw scores and quotients. Using raw scores there is no difference between infected groups, but the difference between infected and uninfected groups persist. This is likely due to the effect of locomotor delay being diluted out in the general score.

The age adjustment may also have had an effect – quotients are calculated in one month periods – age adjustments were performed on actual age and decimals of months. (i.e children with ages 11,2; 11,4; 11,8 months will all have the same quotient but raw scores are age adjusted) (table 5)

**Table 5: Pairwise comparisons between groups of means for age-adjusted raw scores and quotients in each group for locomotor subscale.**

| **Locomotor Subscale** | | | | | | | | | | | |
| --- | --- | --- | --- | --- | --- | --- | --- | --- | --- | --- | --- |
| Time point | Analysis method | ART-Def  Vs  ART-40W | ART-Def  vs  ART -96W | ART-Def  vs  HEU | ART-Def  vs  HU | ART-40W vs  ART-96W | ART-40W vs  HEU | ART-40W vs  HU | ART-96W vs  HEU | ART-96W vs  HU | HEU  vs  HU |
| 1 | Raw | 0.03 | 0.02 | <0.001 | <0.001 | 0.8 | <0.001 | 0.09 | 0.01 | 0.15 | 0.16 |
|  | Quotient | 0.02 | 0.03 | <0.001 | <0.001 | 0.9 | 0.03 | 0.15 | 0.03 | 0.14 | 0.39 |
| 2 | Raw | 0.47 | 0.04 | 0.02 | 0.13 | 0.14 | 0.06 | 0.37 | 0.54 | 0.63 | 0.28 |
|  | Quotient | 0.26 | 0.03 | 0.001 | 0.003 | 0.24 | 0.01 | 0.04 | 0.12 | 0.41 | 0.4 |
| 3 | raw | 0.87 | 0.71 | 0.18 | 0.04 | 0.58 | 0.21 | 0.04 | 0.09 | 0.01 | 0.56 |
|  | Quotient | 0.61 | 0.97 | 0.03 | 0.001 | 0.63 | 0.07 | 0.004 | 0.03 | 0.001 | 0.47 |
| 4 | raw | 0.52 | 0.93 | 0.89 | 0.51 | 0.58 | 0.45 | 0.18 | 0.82 | 0.46 | 0.63 |
|  | Quotient | 0.56 | 0.82 | 0.77 | 0.62 | 0.73 | 0.4 | 0.27 | 0.61 | 0.47 | 0.87 |
| 5 | Raw | 0.35 | 0.57 | 0.88 | 0.06 | 0.73 | 0.45 | 0.33 | 0.68 | 0.21 | 0.1 |
|  | Quotient | 0.44 | 0.5 | 0.59 | 0.12 | 0.96 | 0.85 | 0.43 | 0.9 | 0.42 | 0.35 |

Raw score findings for locomotor subscale confirm that scores are similar at 5 years – both raw scores and quotients comparisons at time point are in agreement with no statistically significant differences (apart from two trends).
